# Towards a Valorization of Corn Bioethanol Side Streams: Chemical Characterization of Post Fermentation Corn Oil and Thin Stillage

**DOI:** 10.3390/molecules25153549

**Published:** 2020-08-03

**Authors:** Gabriella Di Lena, Petra Ondrejíčková, Josè Sanchez del Pulgar, Veronika Cyprichová, Tomáš Ježovič, Massimo Lucarini, Ginevra Lombardi Boccia, Stefano Ferrari Nicoli, Paolo Gabrielli, Altero Aguzzi, Irene Casini, Roberto Caproni

**Affiliations:** 1 CREA Research Centre for Food and Nutrition, Via Ardeatina 546, 00178 Rome, Italy; jose.sanchezdelpulgar@crea.gov.it (J.S.d.P.); massimo.lucarini@crea.gov.it (M.L.); g.lombardiboccia@crea.gov.it (G.L.B.); stefano.nicoli@crea.gov.it (S.F.N.); paolo.gabrielli@crea.gov.it (P.G.); altero.aguzzi@crea.gov.it (A.A.); irene.casini@crea.gov.it (I.C.); roberto.caproni@crea.gov.it (R.C.); 2 ENVIRAL a.s., Trnavská Cesta, 920 41 Leopoldov, Slovakia; Ondrejickova@enviengroup.eu (P.O.); Cyprichova@enviengroup.eu (V.C.); Jezovic@enviral.sk (T.J.)

**Keywords:** corn bioethanol side streams, bioethanol co-products, post-fermentation corn oil, thin stillage, by-products valorization, chemical composition, macronutrients, minerals, fatty acids

## Abstract

First-generation biofuel biorefineries may be a starting point for the development of new value chains, as their by-products and side streams retain nutrients and valuable molecules that may be recovered and valorized for high-value applications. This study provides a chemical characterization of post-fermentation corn oil and thin stillage, side streams of dry-grind corn bioethanol production, in view of their valorization. An overall long-term study was conducted on the two co-products collected over 1 year from a bioethanol plant. Water content, acid value, sedimentation, mineral composition, and fatty acid profiles were analyzed on post-fermentation corn oil. Results highlighted that its acid value was high (19.72–24.29 mg KOH/g), indicating high levels of free fatty acids, but stable over the year due to standardized operating conditions. The fatty acid profile was that typical of corn oil, with a prevalence of linoleic (54–59% of total fatty acids) over oleic (23–27%) and palmitic (12–17%) acids. Macronutrients, fatty acid, and mineral profiles were investigated in thin stillage. Results revealed the acidic pH (4.05–4.68) and high dilution (90–93% water) of this side stream. The dry mass was composed of fats (19–30%), proteins (8.8–12.8%), ash (8.7–9.5%), and fiber (7.3–9.8%). The concomitant presence of a variegate complex of molecules of nutritional interest in corn bioethanol co-products, with several potential high-value market applications, make the perspective of their recovery a promising strategy to create new cross-sector interconnections according to circular economy principles.

## 1. Introduction

With the increasing world population, industrial expansion, and growth of global energy demand, the consumption of fossil fuels has become unsustainable, leading to depletion of resources and global warming.

The promotion of renewable energy is an essential part of the European energy policy, as recognized in the Treaty on the Functioning of the European Union [1] and in the implementation of the Energy Union Strategic Framework [2,3]. The legislative framework for production and usage of biofuels is provided by regulations promoting the use of energy from renewable sources and establishing sustainability criteria in order to reduce greenhouse gas (GHG) emissions and limit global land conversion for biofuel feedstock production [4,5,6]. 

Bioethanol is the second largest contributor of renewable energy sources to the transport sector and represents 20% of biofuels in Europe [7]. It is mainly produced from crop-based feedstock, with corn representing 43% of feedstocks, followed by wheat (26%), sugar-beet (21%), and other cereals and starch-rich crops (6%) [8]. In Europe, the installed grain-to-ethanol production capacity has steadily increased in the last few years, reaching 8901 million L in 2018 with an average of 70% GHG savings compared to fossil fuels [9]. Further increments are expected in the future; yet, to increase the competitiveness of biofuels, a significant reduction of their production costs is necessary. A promising approach is the valorization of co-products and side streams with the creation of biofuel-driven integrated biorefineries, opening opportunities for new bio-based value chains. This approach is in line with the circular economy principles aiming at ensuring high quality, functional, and safe products to all, while reducing carbon and environmental footprints [10,11].

At the same time, with the perspective of a growing world population reaching 9 billion by 2050, sustainable food security, defined as the availability of food providing a healthy and nutritious diet to all, is an emerging issue. Agri-food by-products and agro-industrial wastes, being still rich in nutritious and functional ingredients, should be exploited as sustainable sources of nutrients. Within this context, first-generation biofuel biorefineries may be a starting key point for new sustainable biobased value chains, as their by-products and side streams still retain series of nutrients and valuable molecules that, if properly recovered and valorized, may open new perspectives for integrated biorefinery systems with the concomitant advantage of increasing the competitiveness and maximizing the efficiency of the bio-fuel production process. 

The dry grind corn bioethanol process entails several key steps including enzymatic liquefaction and saccharification, releasing glucose from starch, followed by fermentation by means of the yeast *Saccharomyces cerevisiae* and further distillation. Two investigated co-products of dry grind corn bioethanol production are post-fermentation corn oil and thin stillage. Post fermentation corn oil differs from pressed corn oil by means of corn feedstock being processed by enzymatic hydrolysis followed by a fermentation process and subsequent distillation when being isolated from residual whole stillage by decanter. Post-fermentation corn oil, separated from corn syrup by centrifugation, may be utilized for industrial applications such as bio-diesel production if there is an adjacent biodiesel plant. Prior to this application, the recovery process and valorization of present bioactive compounds (sterols, vitamins, phenolics) should be developed and applied for their high-value applications (food, nutraceuticals, cosmetics). The thin stillage stream is a supernatant liquid fraction generated in large amounts after centrifugation of thick/whole stillage. It consists primarily of suspended and dissolved particles and nutrients originating from spent grains as well as yeast cells; therefore, it is still rich in compounds that deserve to be recovered. Currently, the significant fraction of thin stillage is used to produce animal feed distiller’s dried grains with solubles (DDGS) by multiple energy-intensive evaporations. However, the high cost of concentrating thin stillage and its relatively low nutritional value suggest other applications. One currently applicable use of thin stillage is as a backset water for the liquefaction step preceding fermentation in bioethanol plants [12,13]. Nonetheless, both solutions are low-purpose usages, not really valorizing the full set of components present in it. Alternative strategies to valorize thin stillage have been studied, like the use as a growth medium for filamentous fungi [14,15] or algae [16,17] finalized to obtain high-value biomass and bio-products. Notwithstanding, the extraction and fractionation of valuable compounds present in thin stillage by means of suitable technologies and their utilization as ingredients for high-value markets could represent the most ambitious and profitable way to valorize this side stream.

The EXCornsEED Project, financed within the European Union’s Horizon 2020-BBI-JU Program, has the main goal of developing and validating an integrated process of innovative and highly sustainable technologies to recover proteins and other valuable compounds from side streams of first-generation biofuel biorefineries and to apply them as ingredients for high-value market products. The first step of the Project has been to assess the chemical composition of side streams in order to evaluate their valorization potentials. Post-fermentation corn oil and thin stillage provided by a dry-grind corn bioethanol plant have been the main objects of this study (Figure S1). 

Literature studies by other authors have highlighted the organic and inorganic compounds present in grain-based ethanol thin stillage and post fermentation corn oil [12,18,19,20,21,22,23] but, to our best knowledge, no manuscript reports on a systematic study for an extensive period of time focused on chemical characteristics of co-products of a commercial dry-grind corn-based bioethanol facility. 

We herewith report the results of a study, conducted in the framework of the EXCornsEED Project, focused on the assessment of components of nutritional interest (macronutrients, fatty acids, and minerals) in post-fermentation corn oil and thin stillage collected over a one-year period. The aim of this study was to determine the composition of selected side-streams and to provide comprehensive information about their chemical profile to explore their full valorization potentialities.

## 2. Results and Discussion

### 2.1. Corn Oil

Compositional parameters of the post-fermentation corn oil side stream from bioethanol production play an important part in the subsequent non-edible application for biodiesel production, therefore, corn oil is being routinely tested on a daily basis for output parameters at ENVIRAL premises.

The water residue of vegetable oils is considered a factor diminishing the oil quality and stability because of the physical, chemical, and microbial changes that it may trigger. At the same time, in view of biodiesel production, water is considered an oil contaminant, reducing the yield of the transesterification process [24]. Therefore, the water content together with total impurities are monitored in the oil before its further processing. As shown in Table 1, the levels of water and total contaminants on a monthly basis collected over a one-year period, were below the internal limit of 2% *w*/*w*.

Some parameters, like the sedimentation value, might vary according to seasonal changes, being inversely related to the outer temperature associated to the sedimentation tank surroundings. During the second half of the evaluation year, sedimentation of corn oil was also monitored. From the results shown in Table 1, an increasing trend of the values starting from the summer to winter months was observed. This can be connected with seasonal changes of the outer temperature, which affects the temperature of tanks, where the level of sedimentation in the tanks increases with the lower temperatures.

In line with the high temperatures reached during the biotech process, the acid value of post-fermentation corn oil was quite remarkable (19.72–24.29 mg KOH/g), indicating high levels of free fatty acids. This is a critical parameter, negatively affecting the oxidative stability of the oil and also the yield of the transesterification reaction. In fact, free fatty acids in the oil are converted to soaps during base-catalyzed esterification, thus lowering the yield of biodiesel [25]. The average acid values of corn oil, oscillating in the narrow range 20–25 mg KOH/g, are an indication of stable operating conditions. These values are expected in an industrial oil that underwent severe heat treatments and are comparable to those reported by other authors [22].

Crude vegetable oils contain phosphorus in the form of phospholipids, which are also referred to as gums. As the higher levels of phosphorus in oils have a negative effect on biodiesel yield, this impurity is removed during feedstock pre-treatment in the degumming process. The level of phosphorus is also strictly controlled in the final biodiesel as it increases the exhaust emissions [26]. The results of the average phosphorus content in corn oil confirmed that the values comply with ENVIRAL’s internal limit of 30 mg/kg that corn oil has to meet for its subsequent utilization as a feedstock in an adjacent biodiesel plant. 

Calcium, magnesium, sodium, and potassium are inorganic contaminants that, when present in biodiesel, may negatively affect engine performance and contribute to biodiesel decomposition [27]. As shown in Table 1, the contents of calcium and magnesium were almost constant during the year with fluctuating values of potassium observed but still meeting internal limits and having no impact on final biodiesel product properties. 

The fatty acid profile of individual lots of post-fermentation corn oil is reported in Table 2. The profile observed was that typical of corn oil [28,29], with linoleic acid (C18:2 n-6) as the prevalent fatty acid (54–59% of total fatty acids) followed by oleic (C18:1 n-9, 23–27%) and palmitic (C16:0, 12–17%) acids. Minor amounts of stearic acid (C18:0, 1–2%) and α-linolenic acid (C18:2 n-6, 1.6–2.7%) and trace amounts of other saturated and unsaturated fatty acids were also detected. 

Along with methyl esters, the mass spectrum analysis enabled the identification of minor amounts (5–10%) of ethyl esters of fatty acids like palmitic and linoleic acids, presumably already present in the original sample before transesterification as a result of yeast metabolism. The values reported in Table 2 for palmitic and linoleic acids are the sum of ethyl and methyl esters. The comparison of the different lots of corn oil examined showed a low variability of the fatty acid profile, as indicated from the coefficient of variation (CV) of major compounds (2.5%, 6.1%, and 13.9% for linoleic, oleic, and palmitic acids, respectively). This indicates a comparable quality of corn feedstock and standardized industrial processing conditions. The profile observed is in accordance with that reported by other authors although, to the best of our knowledge, no one has reported on the presence of ethyl esters of fatty acids until now [20].

### 2.2. Thin Stillage 

The proximate composition of different lots of thin stillage collected over the period July 2018-June 2019, expressed on a wet mass and on a dry mass basis, is reported in Table 3. Thin stillage, currently utilized after condensation together with DDGS as a feed ingredient, is characterized by an acidic pH (4.05–4.68) and a very high dilution (about 90–93% water content). pH is within the range of internal limits of pH 3.7–4.7. The acidic pH is mainly attributable to the presence of organic acids, mainly acetic, lactic, and propionic acids resulting from the fermentation process [18,23]. The dry matter value in the series of samples analyzed was almost stable (6.8–9.9%), indicating constant conditions of the biotech process. Nitrogen analyses evidenced high percentages of nonprotein nitrogen (NPN), corresponding to about 50% of total N in all thin stillage batches. NPN compounds in thin stillage arise from corn residues, yeast metabolites, and from the urea added as a N source for yeasts during the bioethanol production process. In consideration of the high NPN content of thin stillage, in place of the common crude protein content calculated on the base of the total N content, that would highly overestimate proteins, we calculated the true protein content, which better reflects the particular composition of thin stillage. 

The dry matter was composed of fats (19–30% dry mass), true proteins (8.8–12.8% dry mass), calculated by subtracting nonprotein N to total N, ash (7.8–11.4% dry mass), and total dietary fiber (8.4–24.4% dry mass). The remaining fraction (36–53% dry mass) may be ascribed to oligosaccharides, unfermented sugars, organic acids, and glycerol left after fermentation, as indicated in the literature [23]. Minor variations observed in the thin stillage composition can be caused by seasonal differences in maize composition and also by slight changes in process parameters such as acid addition rates, enzyme loadings, temperatures, and fermentation conditions.

The mineral and trace element profiles of the different lots of thin stillage analyzed are reported in Table 4. Thin stillage showed a prevalence of potassium (1.8–2.8% dry mass) and phosphorus (1.3–1.8% dry mass) over sodium (0.4–0.9% dry mass) and magnesium (0.5–0.8% dry mass) and minor amounts of calcium (0.06–0.09% dry mass). Iron, zinc, manganese, and copper were detected at very low levels. 

The fatty acid profile of the lots of thin stillage analyzed is reported in Table 5. The profile was similar to that observed for corn oil, with linoleic acid (C18:2 n-6) as the prevalent fatty acid (about 50% of total fatty acids). Palmitic (C16:0) and oleic (C18:1 n-9) acids, accounting altogether for more than 40% of total fatty acids, were the other two most prominent fatty acids. With respect to corn oil, thin stillage showed a higher percentage of saturated fatty acids, mostly contributed by palmitic acid (21–24% of total fatty acids). As also observed in corn oil, in thin stillage, the mass spectrum analysis allowed for the identification of minor amounts of ethyl esters of palmitic and linoleic acids, most probably arising from the fermentation process. The values here reported are the sum of ethyl and methyl esters. Fatty acid values are in accordance with other studies [20], although the presence of ethyl esters was not reported before.

The profile of the different lots of thin stillage examined showed a low variability, as indicated by CV values (<10%), which indicates a uniform quality of the feedstock as well as standardized conditions of the biotechnological process. 

## 3. Materials and Methods

### 3.1. Collection of Side-Stream Samples 

Post-fermentation corn oil and thin stillage samples were obtained from the industrial dry-grind bioethanol plant ENVIRAL a.s. (Leopoldov, Slovakia). A yellow non-genetically modified corn (*Zea mays*), grown in the Central East Europe region, was the feedstock processed. The study was conducted in the period June 2018–June 2019 (Table S1). The first side stream samples (Lot 1) were obtained from corn harvested in the preceding year 2017. The new harvest season started in September 2018 and by October 2018 (Lot 2) the newly harvested corn was utilized for the bioethanol production process. 

Post-fermentation corn oil, obtained by centrifugation of corn syrup, was taken from a plate spinner located under the oil flow control and stored in a sedimentation tank to eliminate any particles that tend to settle and precipitate while cooling down. Thereafter, corn oil samples were put into clean plastic containers and kept in a dry place, away from heat and light sources. 

Thin stillage, currently concentrated by evaporation, was sampled from the discharge valve located in the evaporator compartments of the commercial dry-grind ethanol facility. 

### 3.2. Treatment of Samples

Corn oil samples collected for daily routine analyses (water content, sedimentation, total contamination, acid value, minerals) and thin stillage samples for basic check of pH and dry matter, were immediately taken to the ENVIRAL´s in house laboratory without any further treatment. 

On a monthly basis (approximately every 30–45 days), 1 L of corn oil (liquid) and 3 L of thin stillage (frozen) were delivered (24–48 h from collection) to CREA laboratory (Food and Nutrition Research Centre, Rome, Italy) for in-depth characterization of macronutrients, mineral elements, and fatty acids. Thin stillage samples had been frozen by ENVIRAL straight after sampling, prior to transport, with the intention of preventing any undesirable secondary fermentation during shipment; slow melting during transportation without any changes of thin stillage properties was acceptable, allowing immediate processing at CREA’s lab.

Samples for in-depth chemical characterization, upon arrival at the CREA premises, were immediately refrigerated (+4 °C) and treated as accurately as possible in order to preserve their original properties until analyses. Corn oil was preserved from light and heat and analyzed without any pre-treatment. Before sampling for analyses, the oil was brought to room temperature and gently shaken in order to re-suspend any solid material sedimented at the bottom of the bottle. Thin stillage was analyzed as received, in the liquid state and, when analytical methods required concentrated samples (i.e., fat, minerals, dietary fiber), after lyophilization. The consistency of results obtained on liquid and freeze-dried samples was checked and confirmed by replicate and parallel analyses on freeze-dried and liquid aliquots of thin stillage. Proximate composition, nonprotein nitrogen, total dietary fiber, mineral content, and fatty acid profile were the parameters studied.

### 3.3. Analytical Methods

#### 3.3.1. Daily Routine Analyses 

Water content in corn oil was determined by the Karl-Fisher titration method according to EN ISO 12937 [30]. Sedimentation in corn oil was measured from homogenized sample after centrifugation. The amount of separated material, the sediment, was volumetrically measured in a calibrated centrifuge tube. Total contamination was controlled by gravimetric method according to EN 12662 [31]. A pre-weighed membrane filter was used for the vacuum filtration of a known volume of tested sample, washed, dried, and weighed. Acid value was determined by non-aqueous potentiometric acid-base titration with an alcoholic solution of potassium hydroxide according to EN 14104 [32]. Mineral contents (phosphorus, calcium, magnesium, sodium, and potassium) were determined by inductively coupled plasma (ICP) emission spectrometry according to EN 14107 [33]. 

Thin stillage was routinely checked for pH by potentiometric measurement using a glass electrode and dry matter was determined by weighing after the drying of pre-dried sample in an oven at 130 °C.

#### 3.3.2. Proximate Composition 

Moisture, crude protein, crude fat, and ash contents were determined according to the Association of Official Analytical Chemists’s (AOAC) [34] methods. Total nitrogen was evaluated by the Kjeldahl procedure. Nonprotein nitrogen (NPN) was determined following the Kjeldahl method after protein precipitation with 10% (w/v) trichloroacetic acid and filtration. True protein N was calculated as the difference between total nitrogen and NPN. The true protein content was calculated by multiplying true protein N by the factor 6.25. The crude fat content was determined by Soxhlet extraction. Ash content was determined by incineration in a muffle furnace at 550 °C. Total dietary fiber was determined according to the official enzymatic-gravimetric method [35]. Available carbohydrates, organic acids, and other solubles were calculated by differences. All analyses were performed in triplicate. Results were normalized both on a wet mass and dry mass basis. 

#### 3.3.3. Minerals and Trace Elements 

Minerals (Ca, Mg, Na, K, P) and trace elements (Fe, Zn, Cu, Mn) contents of thin stillage were quantified by inductively coupled plasma optical emission spectrometry (Optima 8000™ ICP-OES, Perkin-Elmer, Waltham, MA, USA) after liquid ashing (6 mL HNO_3_ + 1 mL H_2_O_2_) of lyophilized thin stillage in a microwave digestion system (1200 Mega, Milestone srl, Italy). Standard reference materials, peanut butter (SRM 2387, National Institute of Standards and Technology), cabbage (IAEA-359, International Atomic Energy Agency Reference Materials Group), and haricots vert (BCR 383, Community Bureau of Reference, Brussels), were analyzed as a check on the accuracy of the analysis. Analyses were performed in quadruplicate. Data were normalized both on a wet mass and dry mass basis considering the water content of liquid thin stillage (90–93%). 

#### 3.3.4. Total Lipids and Fatty Acids 

Total lipids were extracted from lyophilized thin stillage samples according to the method of Bligh and Dyer [36]. In both samples of untreated crude corn oil and thin stillage lipid extracts the fatty acids were methylated using boron trifluoride in methanol as esterification reagent [37]. Separations were accomplished on a Mega-wax column (30 m × 0.32 mm inner diameter, 0.25 μm film thickness). The esterified fatty acids were identified and quantified by GC-MS-FID (7890A Series-Agilent Technologies Santa Clara, CA, USA). Fatty acids were identified comparing retention times with known authentic standards and using the NIST08 Mass Spectral Library (National Institute of Standards and Technology, Gaithersburg, MD, USA). FAME Mix C4-C24 (Supelco, Bellofonte, PA, USA) was executed as a control of the accuracy of the analysis. Analyses were performed in triplicate. Data are reported as percent of total fatty acids.

### 3.4. Data Treatment 

For each parameter, analyses of individual lots of post-fermentation corn oil and thin stillage were performed at least in triplicate. Data for single lots, mean, standard deviation, coefficient of variation, and range of values detected during the experimental period were calculated with Microsoft Excel software, 2013 version.

## 4. Conclusions

In conclusion, in this study the complete monthly data, measured during a one-year period, here presented, describe macronutrient, fatty acid and mineral profiles of post-fermentation corn oil and thin stillage from commercial bioethanol production. These data provide basic indications on the main properties and qualities of the two side streams. In particular, the high linoleic and oleic acids concentrations, fatty acids of nutritional interest, and the relatively high and constant presence of minerals and macronutrients in the stillage suggest a biotechnological utilization. The low variability of the chemical characteristics observed during the year in the two side streams, in spite of the different origin and seasonality of corn feedstock lots and of the complex biotechnological processes, represent an important element for their industrial utilization. 

For a full exploitation of the potentialities of the side streams in this study, a detailed investigation on the bioactive compounds present therein and a sustainability assessment of recovery processes represent the next necessary steps of this study. 

The concomitant presence in corn bioethanol co-products of a variegate complex of molecules of nutritional interest, with several different potential high-value market applications, make the perspective of their recovery a promising strategy to maximize the sustainability of corn dry-grind bioethanol biorefineries and create new cross-sector interconnections according to circular economy principles. The aim of the Project is to fractionate the post-fermentation corn oil prior to non-edible biodiesel applications, to obtain desirable valuable molecules, and simultaneously optimize feedstock streams for biodiesel application by removing these molecules.

## Figures and Tables

**Table 1:** Chemical characteristics of corn oil from a dry-grind corn ethanol plant. Data obtained by ENVIRAL on samples collected over a one-year period *.

	Lot 1	Lot 2	Lot 3	Lot 4	Lot 5	Lot 6	Lot 7	Lot 8	Lot 9	mean	sd	min	max
Water content (g/100 g)	0.51 ± 0.07	0.41 ± 0.07	0.45 ± 0.08	0.44 ± 0.07	0.44 ± 0.08	0.34 ± 0.02	0.36 ± 0.03	0.46 ± 0.23	0.43 ± 0.09	0.43	0.05	0.34	0.51
Sedimentation (vol %)	5.88 ± 2.09	7.29 ± 3.96	13.88 ± 4.64	8.8 ± 0.82	7.14 ± 1.89	6.50 ± 2.51	9.20 ± 2.09	11.8 ± 3.24	13.0 ± 2.53	9.28	2.95	5.88	13.88
Total contamination (g/100 g)	0.01 ± 0.01	0.06 ± 0.01	0.01 ± 0.01	0.01 ± 0.01	0.01 ± 0.00	0.01 ± 0.00	0.28 ± 0.03	0.19 ± 0.04	0.01 ± 0.01	0.06	0.10	0.01	0.28
Acid value (mg KOH/g)	21.02 ± 1.26	24.29 ±1.51	21.33 ± 1.91	19.72 ± 0.78	21.79 ± 1.72	20.90 ± 0.79	21.9 ± 1.95	22.86 ± 0.77	20.75 ± 1.21	21.62	1.33	19.72	24.29
Minerals:													
P (mg/kg)	7.5 ± 1.6	6.18 ± 1.08	18.81 ± 11.34	9.78 ± 5.77	34.45 ± 9.44	10.6 ± 2.2	9.75 ± 3.65	3.52 ± 1.28	10.87 ± 2.72	12.38	4.34	3.52	34.45
K (mg/kg)	3.27 ± 1.06	1.61 ± 0.40	11.83 ± 7.42	7.10 ± 2.94	6.33 ± 3.64	<1	7.40 ± 3.58	1.66 ± 0.41	15.53 ± 6.97	-	-	<1	15.53
Na (mg/kg)	0.57 ± 0.31	1.99 ± 1.20	<1	<1	2.57 ± 1.66	2.78 ± 1.66	2.94 ± 1.41	1.02 ± 0.42	4.74 ± 2.53	-	-	0.57	4.74
Mg (mg/kg)	0.84 ± 0.34	<1	<1	<1	1.63 ± 0..47	<1	1.36 ± 0.87	<1	1.37 ±1.17	-	-	0.84	1.63
Ca (mg/kg)	0.05 ± 0.03	<1	<1	<1	<1	<1	<1	<1	<1	-	-	0.05	<1

* data for each monthly lot represent mean ± sd of triplicate measurements. Details on the origin of corn feedstock and on lot timings are provided in Materials and Methods and in Table S1.

**Table 2:** Fatty acid profile of post-fermentation corn oil from the dry-grind corn ethanol plant. Mean, standard deviation, and range of values observed over a one-year period (n = 8) *. Values are expressed as percent of total fatty acids.

	Lot 1	Lot 2	Lot 3	Lot 4	Lot 5	Lot 6	Lot 7	Lot 8	mean	sd	min	max
	% of total fatty acids											
Lauric acid (C12:0)	0.00 ± 0.00	0.02 ± 0.01	0.02 ± 0.01	0.00 ± 0.00	0.00 ± 0.00	0.00 ± 0.00	0.00 ± 0.00	0.00 ± 0.00	0.01	0.01	0.00	0.02
Myristic acid (C14:0)	0.00 ± 0.00	0.04 ± 0.02	0.05 ± 0.02	0.03 ± 0.01	0.08 ± 0.03	0.12 ± 0.03	0.12 ± 0.02	0.00 ± 0.00	0.07	0.04	0.03	0.12
Pentadecylic acid (C15:0)	0.00 ± 0.00	0.00 ± 0.00	0.00 ± 0.00	0.00 ±.0.00	0.00 ± 0.00	0.00 ± 0.00	0.00 ± 0.00	0.09 ± 0.04	0.01	0.03	0.00	0.09
Palmitic acid (C16:0)	12.11 ± 0.30	11.56 ± 0.34	15.11 ± 1.10	14.27 ± 2.13	15.38 ± 0.57	16.31 ± 0.48	17.33 ± 1.85	13.39 ± 1.03	14.43	2.00	11.56	17.33
Palmitoleic acid (C16:1 n-7)	0.00 ± 0.00	0.08 ± 0.00	0.05 ± 0.02	0.05 ± 0.01	0.00 ± 0.00	0.00 ± 0.00	0.00 ± 0.00	0.00 ± 0.00	0.06	0.02	0.05	0.08
Margaric acid (C17:0)	0.04 ± 0.01	0.05 ± 0.01	0.05 ± 0.02	0.00 ± 0.00	0.00 ± 0.00	0.00 ± 0.00	0.00 ± 0.00	0.00 ± 0.00	0.05	0.01	0.04	0.05
Stearic acid (C18:0)	1.40 ± 0.08	1.47 ± 0.09	1.91 ± 0.34	1.84 ± 0.66	1.61 ± 0.05	1.17 ± 0.10	1.06 ± 0.71	1.41 ± 0.03	1.48	0.29	1.06	1.91
Oleic acid (C18:1 n-9)	26.44 ± 1.78	25.99 ± 1.80	23.44 ± 0.43	23.02 ± 0.78	23.13 ± 0.72	25.20 ± 1.32	25.37 ± 2.15	26.85 ± 1.07	24.93	1.53	23.02	26.85
Linoleic acid (C18:2 n-6)	57.99 ± 1.40	58.63 ± 1.41	57.30 ± 0.75	57.06 ± 2.51	57.70 ± 0.29	55.50 ± 0.81	54.12 ± 0.48	56.96 ± 0.27	56.91	1.45	54.12	58.63
α-Linolenic acid (C18:3 n-3)	1.78 ± 0.30	1.78 ± 0.31	1.78 ± 0.38	2.68 ± 1.63	1.98 ± 0.10	1.64 ± 0.09	1.70 ± 0.05	1.73 ± 0.07	1.88	0.34	1.64	2.68
Arachidic acid (C20:0)	0.18 ± 0.05	0.16 ± 0.07	0.07 ± 0.04	0.00 ± 0.00	0.11 ± 0.02	0.00 ± 0.00	0.34 ± 0.08	0.27 ± 0.10	0.19	0.10	0.07	0.34
Gondoic acid (C20:1 n-9)	0.29 ± 0.03	0.21 ± 0.06	0.27 ± 0.02	0.00 ± 0.00	0.18 ± 0.03	0.00 ± 0.00	0.00 ± 0.00	0.00 ± 0.00	0.24	0.05	0.18	0.29
Eicosadienoic acid (C20:2 n-6)	-	-	-	-	-	-	-	-	-	-	-	-
Behenic acid (C22:0)	-	-	-	-	-	-	-	-	-	-	-	-
Total SFA	13.73 ± 0.30	13.27 ± 0.36	17.18 ± 1.10	16.14 ± 1.64	17.13 ± 0.61	17.67 ± 0.49	18.81 ± 2.51	15.14 ± 1.06	16.13	1.95	13.27	18.81
Total MUFA	26.73 ± 1.70	26.26 ± 1.80	23.67 ± 0.53	23.04 ± 0.75	23.19 ± 0.69	25.20 ± 1.32	25.37 ± 2.15	26.85 ± 1.07	25.04	1.56	23.04	26.85
Total PUFA	59.77 ± 1.60	60.40 ± 1.66	59.08 ± 0.64	59.74 ± 0.88	59.67 ± 0.23	57.14 ± 0.90	55.82 ± 0.53	58.12 ± 1.08	58.72	1.57	55.82	60.40
Total n-6 PUFA	57.99 ± 0.50	58.63 ± 1.41	57.30 ± 0.75	57.06 ± 2.51	57.70 ± 0.29	55.50 ± 0.81	54.14 ± 0.48	56.96 ± 0.27	56.91	1.45	54.12	58.63
Total n-3 PUFA	1.78 ± 0.40	1.78 ± 0.31	1.78 ± 0.38	2.68 ± 1.63	1.98 ± 0.10	1.64 ± 0.09	1.70 ± 0.05	1.73 ± 0.07	1.88	0.34	1.64	2.68
n-6/n-3 PUFA ratio	32.58 ± 1.50	33.50 ± 4.79	33.17 ± 6.89	26.54 ± 13.00	29.26 ± 1.58	33.85 ± 1.39	31.81 ± 0.72	32.96 ± 1.02	31.71	2.53	26.54	33.85

* data for each monthly lot represent mean ± sd of triplicate measurements. Details on the origin of corn feedstock and on lot timings are provided in Materials and Methods and in Table S1; - not detected.

**Table 3:** Chemical composition of thin stillage derived from the dry-grind corn ethanol plant. Mean, standard deviation, and range of values observed over a one-year period (n = 9) *.

	Lot 1	Lot 2	Lot 3	Lot 4	Lot 5	Lot 6	Lot 7	Lot 8	Lot 9	mean	sd	min	max
pH	-	4.06 ± 0.11	4.48 ± 0.01	4.22 ± 0.02	4.61 ± 0.07	4.68 ± 0.04	4.48 ± 0.02	4.05 ± 0.01	4.49 ± 0.02	4.38	0.24	4.05	4.68
	g 100 g^−1^ wet mass												
Dry matter	7.69 ± 0.05	7.99 ± 0.05	7.86 ± 0.01	8.44 ± 0.01	6.81 ± 0.07	7.96 ± 0.02	8.74 ± 0.07	9.93 ± 0.08	8.57 ± 0.03	8.22	0.86	6.81	9.93
Water content	92.32 ± 0.02	92.01 ± 0.05	92.14 ± 0.01	91.56 ± 0.01	93.19 ± 0.07	92.04 ± 0.02	91.26 ± 0.07	90.07 ± 0.08	91.43 ± 0.03	91.78	0.86	90.07	93.19
Total N	0.22 ± 0.01	0.23 ± 0.03	0.24 ± 0.01	0.32 ± 0.02	0.27 ± 0.01	0.27 ± 0.01	0.27 ± 0.01	0.32 ± 0.00	0.26 ± 0.01	0.27	0.03	0.22	0.32
Nonprotein N	0.11 ± 0.01	0.12 ± 0.01	0.12 ± 0.01	0.15 ± 0.01	0.13 ± 0.00	0.13 ± 0.01	0.10 ± 0.00	0.13 ± 0.00	0.12 ± 0.00	0.12	0.01	0.10	0.15
True Protein	0.68 ± 0.09	0.71 ± 0.16	0.78 ± 0.01	1.07 ± 0.13	0.87 ± 0.08	0.83 ± 0.08	1.03 ± 0.02	1.19 ± 0.02	0.91 ± 0.03	0.90	0.17	0.68	1.19
Ash	0.73 ± 0.05	0.69 ± 0.02	0.71 ± 0.01	0.75 ± 0.02	0.78 ± 0.01	0.81 ± 0.01	0.73 ± 0.03	0.77 ± 0.02	0.69 ± 0.02	0.74	0.04	0.69	0.81
Crude fat	2.33 ± 0.02	1.52 ± 0.06	1.83 ± 0.02	1.81 ± 0.01	1.46 ± 0.05	1.64 ± 0.02	1.67 ± 0.12	-	-	1.75	0.29	1.46	2.33
Total dietary fiber	0.65 ± 0.08	0.83 ± 0.10	0.95 ± 0.06	1.06 ± 0.01	1.21 ± 0.45	1.31 ± 0.54	2.14 ± 0.08	2.03 ± 0.06	-	1.27	0.54	0.65	2.14
Other solubles **	3.29 ± 0.13	4.24 ± 0.29	3.57 ± 0.11	3.82 ± 0.03	2.51 ± 0.50	3.43 ± 0.60	3.14 ± 0.24	-	-	3.43	0.54	2.51	4.24
	g 100 g^−1^ dry mass												
Dry matter	100	100	100	100	100	100	100	100	100	100			
Total N	2.87 ± 0.11	2.88 ± 0.34	3.10 ± 0.08	3.79 ± 0.21	3.96 ± 0.14	3.35 ± 0.07	3.03 ± 0.10	3.22 ± 0.03	3.07 ± 0.08	3.25	0.39	2.87	3.96
Nonprotein N	1.45 ± 0.10	1.47 ± 0.12	1.50 ± 0.09	1.77 ± 0.07	1.92 ± 0.05	1.68 ± 0.11	1.15 ± 0.05	1.30 ± 0.04	1.38 ± 0.02	1.51	0.24	1.15	1.92
True Protein	8.85 ± 1.22	8.83 ± 2.05	9.99 ± 0.19	12.67 ± 1.58	12.76 ± 1.12	10.46 ± 1.06	11.79 ± 0.28	12.03 ± 0.30	10.59 ± 0.40	10.88	1.51	8.83	12.76
Ash	9.54 ± 0.63	8.64 ± 0.26	9.04 ± 0.12	8.89 ± 0.20	11.40 ± 0.11	10.22 ± 0.08	8.39 ± 0.41	7.75 ± 0.26	8.05 ± 0.21	9.10	1.14	7.75	11.40
Crude fat	30.31 ± 0.14	19.03 ± 0.80	23.33 ± 0.29	21.40 ± 0.05	21.44 ± 0.61	20.57 ± 0.27	19.07 ± 2.01	-	-	22.18	3.88	19.03	30.31
Total dietary fiber	8.41 ± 0.97	10.43 ± 1.30	12.09 ± 1.50	12.56 ± 1.80	17.69 ± 6.72	16.40 ± 6.78	24.43 ± 2.00	20.44 ± 1.50	-	15.31	5.42	8.41	24.43
Other solubles **	42.84 ± 1.67	53.07 ± 3.51	45.42 ± 1.44	45.20 ± 0.25	36.86 ± 7.00	43.05 ± 7.71	35.94 ± 2.56	-	-	43.20	5.77	35.94	53.07

* data for each monthly lot represent mean ± sd of triplicate measurements. Details on the origin of corn feedstock and on lot timings are provided in Materials and Methods and in Table S1; - data not available; ** calculated by difference.

**Table 4:** Minerals and trace element contents of thin stillage from the dry-grind corn ethanol plant. Mean, standard deviation, and range of values observed over a one-year period (n = 9)*.

	Lot 1	Lot 2	Lot 3	Lot 4	Lot 5	Lot 6	Lot 7	Lot 8	Lot 9	mean	sd	min	max
	content per 100 g wet weight												
K (mg)	151.69 ± 2.51	171.55 ± 2.05	172.78 ± 0.57	198.70 ± 3.83	189.29± 5.15	177.84 ± 7.1	196.48 ± 8.15	179.68 ± 1.29	172.20 ± 5.50	178.91	14.52	151.69	198.70
P (mg)	108.78 ± 1.51	111.38 ± 4.1	110.27 ± 1.87	135.10 ± 2.64	126.25 ± 2.0	125.23 ± 1.9	129.17 ± 5.84	126.99 ± 8.64	116.71 ± 4.52	121.10	9.50	108.78	135.10
Na (mg)	47.10 ± 0.52	43.10 ± 0.82	39.71 ±1.53	36.52 ± 0.43	60.49 ± 1.1	66.70 ± 1.66	73.66 ± 3.58	69.74 ± 4.73	47.22 ± 1.59	53.81	13.966	36.52	73.66
Mg (mg)	41.02 ± 0.25	44.55 ± 0.52	41.07 ± 3.79	51.42 ± 1.02	54.04 ± 0.96	51.63 ± 1.47	52.30 ±2.61	48.34 ± 3.66	50.43 ± 1.47	48.31	4.92	41.02	54.04
Ca (mg)	4.72 ± 0.03	5.19 ± 0.26	4.82 ± 0.46	5.71 ± 0.08	6.08 ± 0.11	5.53 ± 0.09	6.50 ± 0.46	7.08 ± 0.55	6.04 ± 0.22	5.74	0.78	4.72	7.08
Fe (mg)	0.66 ± 0.02	0.71 ± 0.02	0.63 ± 0.01	0.82 ± 0.02	0.81 ± 0.02	0.90 ± 0.03	0.85 ± 0.06	0.87 ± 0.07	0.58 ± 0.02	0.76	0.12	0.58	0.90
Zn (mg)	0.59 ± 0.02	0.77 ± 0.03	0.62 ± 0.02	0.75 ± 0.02	0.71 ± 0.01	0.66 ± 0.02	0.75 ± 0.04	0.77 ± 0.05	0.66 ± 0.03	0.70	0.07	0.59	0.77
Mn (mg)	0.22 ± 0.01	0.20 ± 0.01	0.19 ± 0.01	0.22 ± 0.01	0.23 ± 0.01	0.22 ± 0.01	0.24 ± 0.01	0.23 ± 0.02	0.20 ± 0.01	0.22	0.02	0.19	0.24
Cu (mg)	0.02 ± 0.00	0.03 ± 0.00	0.03 ± 0.00	0.03 ± 0.00	0.02 ± 0.00	0.03 ± 0.00	0.03 ± 0.00	0.03 ± 0.00	0.04 ± 0.00	0.03	0.01	0.02	0.04
	content per 100 g dry weight												
K (g)	1.97 ± 0.03	2.15 ± 0.03	2.20 ± 0.09	2.35 ± 0.05	2.78 ± 0.08	2.23± 0.09	2.25 ± 0.09	1.81 ± 0.13	2.01 ± 0.06	2.19	0.27	1.81	2.78
P (g)	1.41 ± 0.02	1.39 ± 0.05	1.40 ± 0.02	1.60 ± 0.03	1.85 ± 0.03	1.57 ± 0.02	1.48 ± 0.07	1.28 ± 0.08	1.36 ± 0.05	1.48	0.17	1.28	1.85
Na (g)	0.61 ± 0.01	0.54 ± 0.01	0.51 ± 0.02	0.43 ± 0.01	0.89± 0.02	0.84 ± 0.02	0.84 ± 0.04	0.70 ± 0.05	0.55± 0.02	0.66	0.17	0.43	0.89
Mg (g)	0.53 ± 0.00	0.56 ± 0.01	0.52 ± 0.05	0.61 ± 0.01	0.79 ± 0.01	0.65± 0.02	0.60 ± 0.03	0.49 ± 0.04	0.59 ± 0.02	0.59	0.09	0.49	0.79
Ca (mg)	61.32 ± 0.40	64.95 ± 3.21	61.32 ± 5.81	67.64 ± 0.94	89.21 ± 1.54	69.46 ± 1.16	74.40 ± 5.24	71.31 ± 5.50	70.50 ± 2.57	70.01	8.46	61.32	89.21
Fe (mg)	8.63 ± 0.21	8.84 ± 0.22	7.96 ± 0.15	9.75 ± 0.24	11.92 ± 0.28	11.26 ± 0.37	9.67 ± 0.68	8.74 ± 0.71	6.76 ± 0.24	9.28	1.59	6.76	11.92
Zn (mg)	7.72 ± 0.21	9.61 ± 0.34	7.95 ± 0.22	8.83 ± 0.19	10.41 ± 0.16	8.27 ± 0.30	8.62 ± 0.42	7.77 ± 0.49	7.69 ± 0.30	8.54	0.94	7.69	10.41
Mn (mg)	2.80 ± 0.04	2.56 ± 0.09	2.48 ± 0.11	2.61 ± 0.06	3.41 ± 0.07	2.80 ± 0.05	2.71 ± 0.13	2.36 ± 0.18	2.36 ± 0.08	2.68	0.32	2.36	3.41
Cu (mg)	0.26 ± 0.01	0.37 ± 0.02	0.35 ± 0.01	0.37 ± 0.01	0.36 ± 0.02	0.36 ± 0.01	0.29 ± 0.02	0.32 ± 0.02	0.51 ± 0.02	0.36	0.07	0.26	0.51

* data for each lot represent mean ± sd of quadruplicate measurements. Details on the origin of corn feedstock and on lot timings are provided in Materials and Methods and in Table S1.

**Table 5:** Fatty acid profile of thin stillage from the dry-grind corn ethanol plant. Mean, standard deviation, and range of values observed over a one-year period (n = 7) *. Values are expressed as percent of total fatty acids.

	Lot 1	Lot 2	Lot 3	Lot 4	Lot 5	Lot 6	Lot 7	mean	sd	min	max
	% of total fatty acids										
Lauric acid (C12:0)	0.00 ± 0.00	0.02 ± 0.00	0.02 ± 0.01	0.00 ± 0.00	0.03 ± 0.00	0.06 ± 0.03	0.00 ± 0.00	0.02	0.02	0.00	0.06
Myristic acid (C14:0)	0.08 ± 0.02	0.06 ± 0.02	0.09 ± 0.00	0.06 ± 0.02	0.12 ± 0.02	0.15 ± 0.04	0.12 ± 0.02	0.10	0.03	0.06	0.15
Pentadecylic acid (C15:0)	0.00 ± 0.00	0.03 ± 0.01	0.02 ±. 000	0.00 ± 0.00	0.00 ± 0.00	0.00 ± 0.00	0.00 ± 0.00	0.01	0.01	0.00	0.03
Palmitic acid (C16:0)	23.29 ± 0.30	23.00 ± 0.37	22.12 ± 0.70	24.10 ± 0.47	23.28 ± 0.38	22.17 ± 2.39	21.40 ± 0.66	22.76	0.91	21.40	24.10
Palmitoleic acid (C16:1 n-7)	0.00 ± 0.00	0.09 ± 0.03	0.06 ± 0.01	0.05 ± 0.00	0.07 ± 0.01	0.00 ± 0.00	0.00 ± 0.00	0.04	0.04	0.00	0.09
Margaric acid (C17:0)	0.00 ± 0.00	0.07 ± 0.00	0.10 ± 0.01	0.00 ± 0.00	0.00 ± 0.00	0.00 ± 0.00	0.00 ± 0.00	0.03	0.05	0.00	0.10
Stearic acid (C18:0)	3.31 ± 0.07	3.16 ± 0.08	3.32 ± 0.03	3.48 ± 0.41	2.49 ± 0.06	1.37 ± 0.19	1.77 ± 0.14	2.70	0.85	1.37	3.48
Oleic acid (C18:1 n-9)	21.08 ± 0.50	21.35 ± 0.54	21.20 ± 0.67	20.36 ± 1.10	21.13 ± 0.17	24.79 ± 1.42	23.73 ± 0.47	21.95	1.64	20.36	24.79
Linoleic acid (C18:2 n-6)	49.14 ± 0.30	49.79 ± 0.32	50.76 ± 0.17	49.48 ± 1.07	50.55 ± 0.29	49.35 ± 1.32	50.95 ± 0.05	50.00	0.74	49.14	50.95
α-Linolenic acid (C18:3 n-3)	1.75 ± 0.05	1.82 ± 0.05	1.63 ± 0.08	1.92 ± 0.15	1.65 ± 0.04	1.40 ± 0.11	1.75 ± 0.05	1.70	0.17	1.40	1.92
Arachidic acid (C20:0)	0.16 ± 0.02	0.32 ± 0.02	0.34 ± 0.02	0.25 ± 0.07	0.30 ± 0.03	0.26 ± 0.04	0.29 ± 0.05	0.27	0.06	0.16	0.34
Gondoic acid (C20:1 n-9)	0.39 ± 0.04	0.18 ± 0.11	0.28 ± 0.07	0.00 ± 0.00	0.26 ± 0.03	0.41 ± 0.04	0.00 ± 0.00	0.25	0.15	0.00	0.41
Eicosadienoic acid (C20:2 n-6)	0.00 ± 0.00	0.07 ± 0.01	0.09 ± 0.03	0.29 ± 0.08	0.00 ± 0.00	0.00 ± 0.00	0.00 ± 0.00	0.08	0.11	0.00	0.29
Behenic acid (C22:0)	0.00 ± 0.00	0.13 ± 0.01	0.13 ± 0.00	0.00 ± 0.00	0.14 ± 0.02	0.06 ± 0.04	0.00 ± 0.00	0.09	0.06	0.00	0.14
Total SFA	26.84 ± 0.50	26.69 ± 0.52	26.03 ± 0.70	27.90 ± 0.90	26.35 ± 0.45	24.06 ± 2.66	23.57 ± 0.46	25.92	1.56	23.57	27.90
Total MUFA	21.47 ± 0.40	21.63 ± 0.43	21.53 ± 0.60	20.41 ± 1.10	21.46 ± 0.17	25.19 ± 1.38	23.73 ± 0.47	22.20	1.65	20.41	25.19
Total PUFA	50.89 ± 0.30	51.69 ± 0.28	52.48 ± 0.26	51.69 ± 1.11	52.20 ± 0.32	50.75 ± 1.43	52.70 ± 0.02	51.77	0.75	50.75	52.70
Total n-6 PUFA	49.14 ± 0.30	49.87 ± 0.32	50.85 ± 0.18	49.77 ± 1.11	50.55 ± 0.29	49.35 ± 1.32	50.95 ± 0.05	50.07	0.72	49.14	50.95
Total n-3 PUFA	1.75 ± 0.05	1.82 ± 0.05	1.63 ± 0.08	1.92 ± 0.15	1.65 ± 0.04	1.40 ± 0.11	1.75 ± 0.05	1.70	0.17	1.40	1.92
n-6/n-3 PUFA ratio	28.08 ± 0.70	27.43 ± 0.88	31.29 ± 1.42	26.01 ± 2.26	30.61 ± 0.69	35.35 ± 1.84	29.07 ± 0.82	29.69	3.08	26.01	35.35
PUFA/SFA ratio	1.90 ± 0.05	1.94 ± 0.05	2.02 ± 0.06	1.85 ± 0.09	1.98 ± 0.05	2.13 ± 0.31	2.24 ± 0.04	2.01	0.14	1.85	2.24

* data for each lot represent mean ± sd of triplicate measurements. Details on the origin of corn feedstock and on lot timings are provided in Materials and Methods and in Table S1.

## Supplementary Materials

The following are available online, Figure S1: Scheme of grain to ethanol process and its connection to the EXCornsEED project. Table S1: Sampling date at ENVIRAL’s plants of the different side stream lots analysed in the study.
