# Supplementary material for: Towards a Valorization of Corn Bioethanol Side Streams: Chemical Characterization of Post Fermentation Corn Oil and Thin Stillage
Source: Molecules. 2020 Aug 3;25(15):3549. doi: 10.3390/molecules25153549 (PMC7435661; doi:10.3390/molecules25153549)
Supplement: Supplementary file 1 [file molecules-25-03549-s001.pdf]

Supplementary information

Towards a Valorization of Corn Bioethanol Side Streams: Chemical Characterization of Post Fermentation Corn Oil and Thin Stillage

Gabriella Di Lena [1,*], Petra Ondrejíčková [2], Josè Sanchez del Pulgar [1], Veronika Cyprichová [2], Tomáš Ježovič [2], Massimo Lucarini [1], Ginevra Lombardi Boccia [1], Stefano Ferrari Nicoli [1], Paolo Gabrielli [1], Altero Aguzzi [1], Irene Casini [1], Roberto Caproni [1]

[1] CREA Research Centre for Food and Nutrition, Via Ardeatina 546, 00178 Rome, Italy; jose.sanchezdelpulgar@crea.gov.it (J.S.d.P); massimo.lucarini@crea.gov.it (M.L.); g.lombardiboccia@crea.gov.it (G.L.B.);

[2] ENVIRAL a.s., Trnavská cesta, 920 41 Leopoldov, Slovak Republic; Ondrejickova@enviengroup.eu (P.O.)

* Correspondence: gabriella.dilena@crea.gov.it; Tel.: +39-06-51494445

Figure S1. Scheme of grain to ethanol process and its connection to the EXCornsEED project.

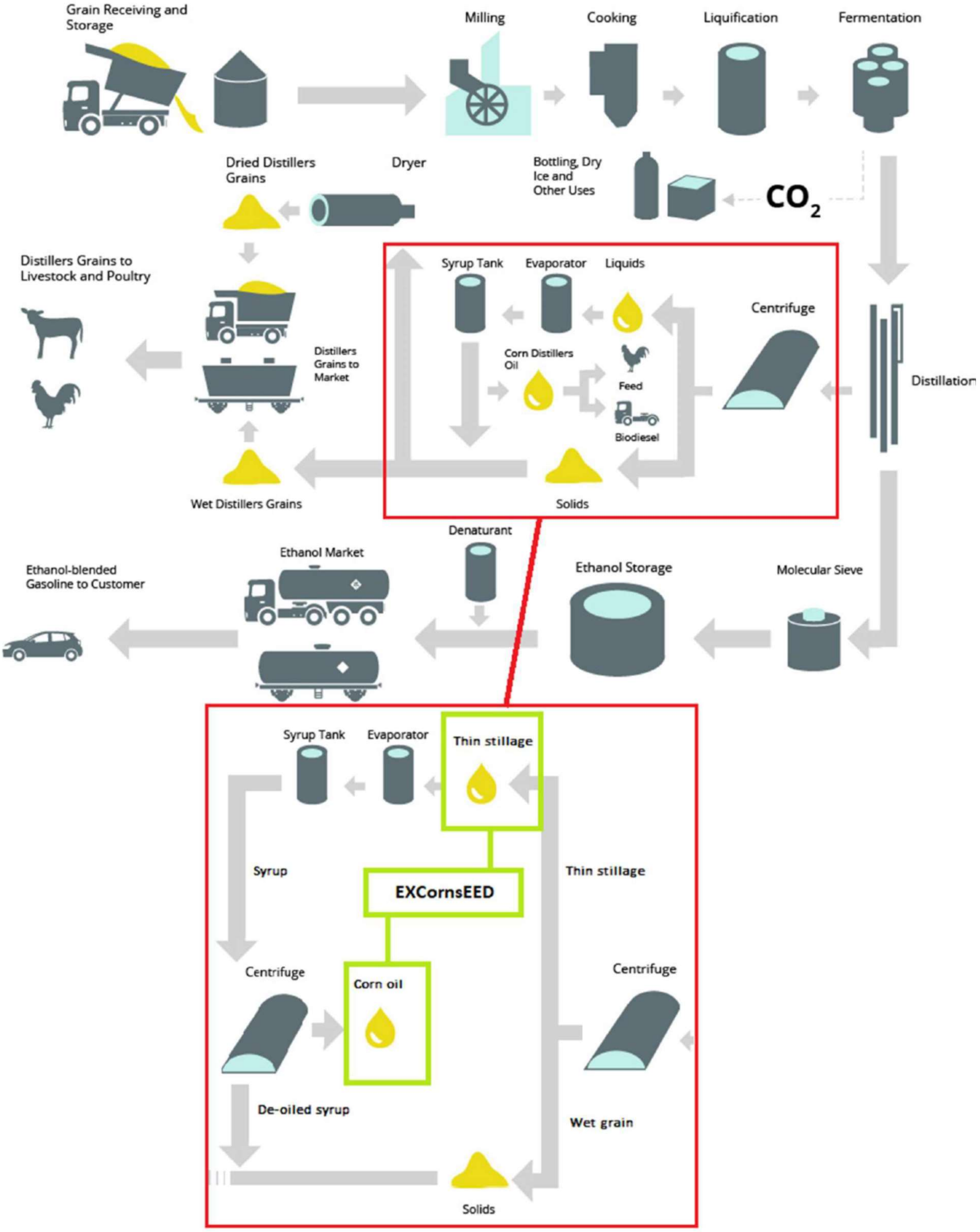

Table S1. Sampling date at ENVIRAL's plants of the different side stream lots analysed in the study.

	SAMPLING DATE	
**LOT**	CORN OIL	THIN STILLAGE
**1**	29/6/2018	29/6/2018
**2**	22/10/2018	22/10/2018
**3**	26/11/2018	26/11/2018
**4**	9/12/2018	5/12/2018
**5**	18/1/2019	18/1/2019
**6**	25/2/2019	25/2/2019
**7**	22/4/2019	18/4/2019
**8**	23/5/2019	24/5/2019
**9**	21/6/2019	21/6/2019
